# Is a high serum copper concentration a risk factor for implantation failure?

**DOI:** 10.1186/s13104-017-2708-4

**Published:** 2017-08-10

**Authors:** Hidehiko Matsubayashi, Kotaro Kitaya, Kohei Yamaguchi, Rie Nishiyama, Yukiko Takaya, Tomomoto Ishikawa

**Affiliations:** Reproduction Clinic Osaka, 15F, Grand Front Osaka Tower A, 4-20 Ofukacho, Kita, Osaka, 530-0011 Japan

**Keywords:** Copper, Implantation failure, Nutrition, Placentation, Wilson’s disease, Zinc

## Abstract

**Background:**

Copper-containing contraceptive devices may deposit copper ions in the endometrium, resulting in implantation failure. The deposition of copper ions in many organs has been reported in patients with untreated Wilson’s disease. Since these patients sometimes exhibit subfertility and/or early pregnancy loss, copper ions were also considered to accumulate in the uterine endometrium. Wilson’s disease patients treated with zinc successfully delivered babies because zinc interfered with the absorption of copper from the gastrointestinal tract. These findings led to the hypothesis that infertile patients with high serum copper concentrations may have implantation failure due to the excess accumulation of copper ions. The relationship between implantation (pregnancy) rates and serum copper concentrations has not yet been examined. The Japanese government recently stated that actual copper intake was higher among Japanese than needed. Therefore, the aim of the present study was to investigate whether serum copper concentrations are related to the implantation (pregnancy) rates of human embryos in vivo.

**Methods:**

We included 269 patients (age <40 years old) who underwent vitrifying and warming single embryo transfer with a hormone replacement cycle using good blastocysts (3BB or more with Gardner’s classification). Serum hCG, copper, and zinc concentrations were measured 16 days after the first date of progesterone replacement. We compared 96 women who were pregnant without miscarriage at 10 weeks of gestation (group P) and 173 women who were not pregnant (group NP).

**Results:**

No significant differences were observed in age or BMI between the groups. Copper concentrations were significantly higher in group NP (average 193.2 μg/dL) than in group P (average 178.1 μg/dL). According to the area under the curve (AUC) on the receiver operating characteristic curve for the prediction of clinical pregnancy rates, the Cu/Zn ratio (AUC 0.64, 95% CI 0.54–0.71) was a better predictor than copper or zinc. When we set the cut-off as 1.59/1.60 for the Cu/Zn ratio, sensitivity, specificity, the positive predictive value, and negative predictive value were 0.98, 0.29, 0.71, and 0.88, respectively.

**Conclusions:**

Our single-center retrospective study suggests that high serum copper concentrations (high Cu/Zn ratio) are a risk factor for implantation failure.

## Background

Copper-containing contraceptive devices have been suggested to deposit copper ions in the endometrium, resulting in implantation failure [1–3]. The deposition of copper ions in many organs, particularly the liver, brain, and eyes has been reported in patients with untreated Wilson’s disease [4]. Since these patients sometimes exhibit subfertility and/or early pregnancy loss [5–7], copper ions were also considered to accumulate in the uterine endometrium. In order to achieve live births, patients with Wilson’s disease have been treated with d-penicillamine, which binds to copper, resulting in its excretion in urine [5–11]. A recent study reported that Wilson’s disease patients treated with zinc successfully delivered babies [12] because zinc interfered with the absorption of copper from the gastrointestinal tract. These findings led to the hypothesis that infertile patients with high serum copper concentrations may have implantation failure due to the excess accumulation of copper ions. According to recent food intake reports in Japan [13–15], actual copper intake (0.97 mg/day) was higher than that needed (0.90 mg/day), whereas actual zinc intake (6.5 mg/day) was lower (10.0 mg/day) among Japanese women aged 30–49 years. Therefore, we hypothesized that pregnancy rates (implantation failure) may be reduced among Japanese women without Wilson’s disease or even women who have not used copper-containing contraceptive devices because of high serum copper (low zinc) concentrations. To the best of our knowledge, the relationship between implantation (pregnancy) rates and serum copper concentrations has not yet been examined. Therefore, the aim of the present study was to investigate whether serum copper concentrations are related to the implantation (pregnancy) rates of human embryos in vivo without Wilson’s disease or copper-containing contraceptive devices.

## Methods

A single-center retrospective case–control pilot study was performed. All patients provided written informed consent for the study, because our study involved the use of human data. Institutional Review Board (IRB) approval was obtained from Jinjukai (mother organization of Reproduction Clinic Osaka).

In our institute, IVF or ICSI was performed with the freeze-all plan (i.e., not performing fresh embryo transfer), followed by thawing embryo transfer in most patients, regardless of ovarian stimulation protocols (i.e., controlled-ovarian stimulation, mild stimulation, and natural cycle). In a controlled-ovarian stimulation, FSH (Gonal F, Merk Serono, Darmstadt, Germany) or HMG (HMG Ferring, Ferring Pharmaceuticals, Lausanne, Switzerland; HMG Fuji, Fuji Pharma, Tokyo, Japan) was administered every day from the 3rd day of menstruation to 2 days before oocyte retrieval. The dose administered (150–450 IU) and combination of FSH/HMG depended on the woman’s ovarian reserve (as tested by anti-Mullerian hormone levels, the basal antral follicle count, and basal FSH level). A GnRH antagonist (Cetrotide at 0.25 mg, Merck Serono) was administered when at least one follicle reached >14 mm. When more than two follicles were >18 mm, ovulation was triggered with hCG (HCG Mochida, Mochida Pharmaceutical, Tokyo, Japan) and cumulus-oocyte complexes were retrieved transvaginally 36 h later. After semen was collected manually within 2 h before oocyte retrieval, sperm was extracted with Sperm Preparation Medium (10700060A, Origio a/s, Måløv, Denmark) or Supra Sperm (10970060J, Origio a/s) depending on the condition of sperm.

Fertilization was performed by IVF and/or ICSI depending on the condition of sperm, and cultured in fertilization media (Universal IVF Medium 10310060A, Origio a/s), followed by blastocyst media (global total LGGT-30, LifeGlobal, Canada) at 37 °C in benchtop incubators (Benchtop Incubator BT-37, Origio a/s) under 5% O_2_, 6% CO_2_, and 89% N_2_ (mixed gas). Embryos were vitrified with vitrification media (VT507#91137, KITAZATO BioPharma, Shizuoka, Japan), placed on a cryotop (KITAZATO BioPharma), and then stored in liquid nitrogen. At the time of warming, thawing media (VT508#91147, KITAZATO BioPharma) was used. Vitrifying and warming methods were performed according to the manufacturer’s manual. All warming embryos were subjected to assisted hatching, which was performed under an inverted microscope using the ZILOS-tk Zona Infrared Laser Optical System (Hamilton Thorne Instruments Biosciences, Beverly, MA, USA). Regarding warming embryo transfer (ET), patients started the oral administration of conjugated estradiol (premarin, 0.625 mg × 6 Tab/day, Pfizer Japan, Tokyo, Japan), and were assessed weekly until an endometrial thickness of ≥8 mm with serum estradiol (E2) ≥140 pg/mL and serum progesterone (P4) < 1.0 ng/mL was reached. If P4 ≥1.0 ng/mL, the embryo transfer cycle was canceled. Progesterone was administered intramuscularly (progesterone, 50 mg/day, Fuji Pharma) and/or transvaginally (utrogestan, 200 mg × 4 tabs/day, Fuji Pharma) depending on patient convenience. If serum E2 < 140 pg/mL or P4 < 10 ng/mL was observed on the day of transfer, additional medications were supplemented until they were greater than these levels. The transfer of blastocysts was performed after 5 days (day 5) of supplementation with progesterone (day 0).

**Fig. 1:**
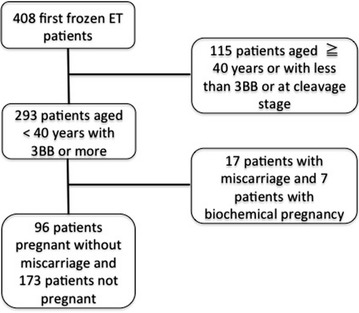
Selection of cases and controls

A total of 408 patients, who underwent the first vitrifying and warming embryo transfer between December 2014 and March 2015, were included in the present study. Patients aged <40 years old and with a good blastocyst (3BB or more with Gardner’s classification) transfer cycle were retrospectively selected for analyses. Pregnancy was defined as confirming gestational sac in the uterus. Miscarriage was defined as a missing or undetectable heart beat at 10 weeks of gestation, while being pregnant without miscarriage was defined as a confirmed heart beat at 10 weeks of gestation. Ninety-six women who were pregnant without miscarriage at 10 weeks of gestation (group P) and 173 women who were not pregnant (group NP) were compared. In order to reduce potential sources of bias, we selected patients with a low risk of aneuploidy, with only the first embryo transfer attempt, and with the same hormone replacement protocol and excluded biochemical pregnancies and miscarriages. The selection of cases and controls was shown in Fig. 1. All patients were followed-up completely without any missing data. Ethnicity was Japanese only. Age, body mass index (BMI), E2, P4, and human chorionic gonadotropin (hCG) were assessed as possible confounders.

Serum hCG, E2, P4, copper, zinc, and ceruloplasmin concentrations were measured 16 days (day 16) after the first date of progesterone replacement (day 0) after at least 2 h of fasting because a pregnancy test was performed on the 16th day in our protocol. E2 and P4 were assessed using a solid-phase, competitive, enzyme immunoassay (iE2 and PROG2, Tosoh Co., Tokyo, Japan), while hCG was analyzed by a solid-phase, sandwich, enzyme immunoassay (HCG2, Tosoh Co.) with an AIA-2000 analyzer (Tosoh Co.). Copper, zinc, and ceruloplasmin concentrations were measured by a clinical laboratory company (Medic Co., Shiga, Japan) using the 4-(3,5-dibromo-2-pyridylazo)-*N*-ethyl-*N*-(3-sulfopropyl) aniline method, 2-(5-nitro-2-pyridylazo)-5-(*N*-propyl-*N*-sulfopropylamino) phenol method, and nephrometry method, respectively.

The Student’s *t* test (non-homogeneity, two-sided) was used for comparisons between two groups. The Chi squared test was used for a 2 × 2 contingency table. Correlations between two groups were detected by Pearson’s test. Receiver operating characteristic (ROC) curves were constructed to demonstrate the predictive accuracy of parameters as a single predictor. The corresponding area under the curve (AUC) was calculated and cut-off values were established as prediction models for the prediction of clinical pregnancy rates (CPR). Better cut-off values for predicting CPR were calculated with sensitivity, specificity, the positive predictive value (PPV), and negative predictive value (NPV). Significance was defined as P < 0.05. The appropriateness of these statistical tests was confirmed by a statistician.

## Results

Table 1 shows the demographic features and clinical parameters of the two groups. No significant differences were observed in age, BMI, E2, or P4 between group NP and group P. Copper concentrations (mean ± standard deviation) were significantly higher in group NP (193.2 ± 31.4 μg/dL) than in group P (178.1 ± 34.4 μg/dL), while zinc concentrations were significantly lower in group NP (75.5 ± 14.1 μg/dL) than in group P (90.7 ± 38.8 μg/dL). When we calculated the copper/zinc ratio (Cu/Zn), it was significantly higher in group NP (2.66 ± 0.71) than in group P (2.23 ± 0.83). All patients had normal ceruloplasmin levels, denying Wilson’s disease. None of the patients used copper-containing contraceptives.

**Table 1:** Demographic features and clinical parameters between two groups

	NP (n = 173)	P (n = 96)	P value
Age (years)	34.8 ± 3.8	34.1 ± 4.0	NS
BMI (kg/m^2^)	21.0 ± 2.6	21.2 ± 3.5	NS
Cu (μg/dL)	193.2 ± 31.4	178.1 ± 34.4	0.0004
Zn (μg/dL)	75.5 ± 14.1	90.7 ± 38.8	0.0003
Cu/Zn ratio	2.66 ± 0.71	2.23 ± 0.83	0.00003
E2 (pg/mL)	271 ± 167	302 ± 168	NS
P4 (ng/mL)	13.1 ± 4.9	13.2 ± 4.7	NS
hCG (IU/L)	0.3 ± 1.1	555 ± 488	<0.00001
Ceruloplasmin (mg/dL)	39.6 ± 6.0	NT	–

Patients who were at the first freeze and thaw embryo transfer attempt with good blastocysts (3BB or more with Gardner’s classification) and who were <40 years old were included. Values indicate the mean ± standard deviation. The Student’s *t* test (non-homogeneity, two-sided) was used for comparisons between two groups

*NP* not pregnant, *P* pregnant without miscarriage at 10 weeks of gestation, *NS* not significant, *NT* not tested

According to the AUC on the ROC curve for the prediction of CPR, the Cu/Zn ratio (AUC 0.64, 95% CI 0.54–0.71) was a better predictor than copper or zinc (Table 2). When we set the cut-off as 1.59/1.60 for the Cu/Zn ratio, sensitivity, specificity, PPV, and NPV were 0.98, 0.29, 0.71, and 0.88, respectively (Table 3).

**Table 2:** AUC of prediction models for the prediction of clinical pregnancy rates

Parameter	AUC	95% CI
Copper	0.60	0.52–0.67
Zinc	0.59	0.51–0.66
Cu/Zn ratio	0.64	0.54–0.71

*AUC* area under the curve, *CI* confidence interval

**Table 3:** Cut-off values for the prediction of clinical pregnancy rates

Parameter	Cut-off	Sensitivity	Specificity	PPV	NPV
Copper	159/160	0.88	0.26	0.68	0.57
Zinc	89/90	0.88	0.34	0.71	0.62
Cu/Zn ratio	1.59/1.60	0.98	0.29	0.71	0.88

*PPV* positive predictive value, *NPV* negative predictive value

Serum copper concentrations were not associated with age, BMI, zinc, E2, P4, or hCG (data not shown), which were thought to be potential confounders.

## Discussion

Our results suggest, for the first time, that high serum copper (low zinc) concentrations are related to implantation failure.

Copper-containing contraceptive devices are considered to deposit copper ions in the endometrium, resulting in implantation failure [1–3]. Copper has been shown to accumulate in the liver, brain, and eyes of patients with Wilson’s disease, a rare autosomal recessive disorder (1/50,000–1/100,000), causing neurological, psychiatric, and liver diseases [4]. Since these patients sometimes exhibit subfertility and/or early pregnancy loss [5–7], copper ions are also considered to accumulate in the uterine endometrium. In order to achieve live births, patients with Wilson’s disease have been treated with D-penicillamine, which binds to copper, resulting in its excretion in urine [5–11]. A recent study reported that Wilson’s disease patients treated with zinc delivered babies successfully [12] because zinc interfered with the absorption of copper from the gastrointestinal tract. These findings suggest that the deposition of copper ions in the uterine endometrium causes infertility and/or early pregnancy loss. According to recent food intake reports in Japan [13–15], actual copper intake (0.97 mg/day) was higher than that needed (0.90 mg/day), whereas actual zinc intake (6.5 mg/day) was lower (10.0 mg/day) among Japanese women aged 30–49 years. Our results support the hypothesis that pregnancy rates (implantation failure) may be reduced among Japanese women without Wilson’s disease or even women who have not used copper-containing contraceptive devices because of high serum copper (low zinc) concentrations.

A copper-containing intrauterine device releases copper into the uterus for 4 years after its insertion [2]. Copper accumulates, even in the epithelium of the tubal tissue, with the insertion of a copper-containing intrauterine device, but not in the serum [3]. Patients with Wilson’s disease have extremely low levels of ceruloplasmin, which binds to copper, resulting in low serum copper concentrations, but high copper concentrations in urine and tissues. The inhibition of copper absorption by zinc is related to competition for binding sites on metallothionein in intestinal mucosal cells [16]. Metallothionein binds copper, zinc, and cadmium ions, while copper is the most tightly bound ion and may displace other ions. Therefore, supplementation with zinc effectively reduces serum copper concentrations because of competitive absorption between copper and zinc. As a result, serum copper and zinc concentrations are inverse proportionally.

Copper and zinc are essential trace elements, with deficiencies or excesses impairing cell functions and affecting cell growth, development, the immune system, and metabolism [17, 18]. Copper and zinc are involved in antioxidative enzymes (superoxide dismutases) in the cytosol. Recent studies indicated that an elevated Cu/Zn ratio was associated with increased oxidative stress [19], several cancers including in the uterine endometrium [20, 21], and the pathogenesis of preeclampsia [22]. Another study also indicated that the Cu/Zn ratio was a more reliable indicator for vascular complications of preeclampsia [23]. Similarly, pregnancies in patients with Wilson’s disease have sometimes been complicated with preeclampsia and/or HELLP syndrome [24, 25]. These findings suggest that patients with high Cu/Zn ratios may have abnormal placentation and the unsuccessful invasion of trophoblasts in the uterine endometrium.

Komai [15] previously reported that taste disturbances caused by a zinc deficiency were more common in Japan than in Western countries. The reason Japanese women had low zinc (high copper) concentrations may be partly explained by their eating habits; they may eat more soybeans and processed foods than meat. Meat is one of the best sources of zinc, soybeans contain phytic acid, a natural zinc-chelating agent, and processed foods contain many food additives, which function as zinc-chelating agents (c.f., EDTA, polyphosphoric acid, and carboxymethyl cellulose) [26]. Recent national food balance data obtained from 176 countries indicated that approximately 20.5% of the world’s population (33.1% in Southeast Asia) is estimated to be at risk of an inadequate zinc intake [27]. Thus, we recommend all women who do not have Wilson’s disease and those who have not used copper-containing contraceptive devices to pay more attention to their food and eating habits (i.e., more meat, less soybeans, and less processed foods), which may be altered.

Limitations that may have contributed to our results include the following. Since we did not show copper deposition in the uterus in the present study, high serum copper (low zinc) concentrations may not be related to the intrauterine accumulation of copper. However, a previous study reported that metals easily accumulate in the uterine endometrium [28]. Furthermore, transferred embryos may include aneuploidy. We were unable to perform preimplantation genetic screening (PGS) because it is not permitted in Japan. However, we selected patients with a low risk of aneuploidy who were <40 years old and had good blastocysts (3BB or more). In order to exclude patients with poor prognoses or to reduce different ovarian functions among patients, only the first embryo transfer attempt was included and the same hormone replacement protocol was performed, and we also excluded biochemical pregnancies and miscarriages. Another limitation is that since our study was retrospective, only associations and not causations between high Cu/Zn ratios and implantation failure may be inferred from the results obtained. Since this study was performed on a narrow study population (women in a single center around the Osaka area in Japan), the results obtained may not apply to women in other locations because a previous study indicated that copper intake by women living in rural areas was higher than that by those in urban areas in Japan [29].

## Conclusions

This was the first study to investigate whether high serum copper (low zinc) concentrations are a risk factor for implantation failure. However, this was from a narrow patient group in a single center retrospective study. A prospective study measuring copper and/or zinc concentrations before hormone replacement cycles and a randomized controlled study on the efficacy of zinc supplementation in patients with high Cu/Zn ratios are required and currently underway in our institute.

## Abbreviations

AUC: area under the curve
BMI: body mass index
CI: confidence interval
CPR: clinical pregnancy rate
Cu/Zn: copper/zinc ratio
E2: estradiol
group NP: non-pregnant group
group P: pregnant group
hCG: human chorionic gonadotropin
HELLP syndrome: hemolysis, elevated liver enzymes, and low platelet count syndrome
NPV: negative predictive value
P4: progesterone
PGS: preimplantation genetic screening
PPV: positive predictive value
ROC curve: receiver operating characteristic curve
